# Testicular Infarction and Rupture as an Uncommon Complication of Epididymo-Orchitis: A Case Report

**DOI:** 10.7759/cureus.110380

**Published:** 2026-06-07

**Authors:** Rakan Aldarrab, Alhassan Almonawar, Abdulrahman Alsayyari, Khalid Albawardi

**Affiliations:** 1 Department of Urology Surgery, King Abdulaziz Medical City, Ministry of National Guard Health Affairs, Riyadh, SAU

**Keywords:** acute scrotum, case report, epididymo-orchitis, multidrug-resistant escherichia coli, orchiectomy, scrotal doppler ultrasound, testicular infarction, testicular rupture

## Abstract

We describe an unusual case of testicular infarction with tunica albuginea rupture occurring as a complication of multidrug-resistant *Escherichia coli* epididymo-orchitis in a 56-year-old man with poorly controlled type 2 diabetes mellitus. Clinical examination and routine laboratory testing cannot reliably distinguish complicated from uncomplicated epididymo-orchitis, and Doppler ultrasound is the key diagnostic tool. The patient presented with one week of progressive left scrotal pain and swelling that had failed to improve on outpatient oral antibiotic therapy. Examination demonstrated marked left hemiscrotal swelling with an absent cremasteric reflex; a tense, reactive hydrocele precluded testicular palpation. Scrotal ultrasound showed an enlarged, diffusely hypoechoic left testis (3.7 × 3.2 × 5.2 cm) with completely absent intratesticular Doppler flow, a large septated hydrocele, and a congested spermatic cord. Emergency scrotal exploration revealed turbid, foul-smelling fluid, breach of the tunica albuginea with extruded seminiferous tubules, and a non-viable dusky testis with no demonstrable flow on intraoperative Doppler, prompting left orchiectomy. Cultures grew multidrug-resistant *Escherichia coli* sensitive only to carbapenems. Recovery was uneventful. This case underscores that refractory or rapidly progressive epididymo-orchitis, particularly in patients with diabetes or other immunocompromising conditions, should prompt urgent re-imaging with Doppler ultrasound and a low threshold for surgical exploration, since absent intratesticular flow and tunica albuginea disruption mandate immediate operative intervention to prevent sepsis and to confirm tissue viability.

## Introduction

Acute scrotal pain is a common presentation in emergency departments and primary care, with epididymo-orchitis among the most frequent diagnoses in adult men beyond the second decade of life [1]. The condition most often arises from retrograde ascent of urinary pathogens from the urethra or bladder [1,2]. The dominant pathogens are sexually transmitted organisms (*Chlamydia trachomatis*, *Neisseria gonorrhoeae*) in younger men and *Enterobacterales*, notably *Escherichia coli*, in men older than 35 years and in those with bladder outflow obstruction or recent genitourinary instrumentation [1,2]. Diagnosis is largely clinical, supported by urinalysis and scrotal Doppler ultrasound; the latter is the preferred imaging modality in the acute setting [2,3]. The mainstay of treatment is empirical antibiotic therapy guided by local microbiology data, with most uncomplicated cases responding within several days [1].

Progression to testicular infarction is uncommon and has been described primarily in case reports and small series [4-9]. The proposed mechanism, articulated by Hourihane more than five decades ago and reinforced in subsequent reports, is one of inflammation-driven venous outflow obstruction: edema of the epididymis, spermatic cord, and surrounding soft tissues, together with an enlarging pyocele, raises pressure within the closed tunica albuginea compartment beyond venous pressure and ultimately beyond arterial perfusion pressure, producing ischemic necrosis [4,5,9,10]. When this process advances, breach of the tunica albuginea (testicular rupture) can happen, although this is rare in a non-traumatic setting and has been documented only in isolated case reports [4,7].

Several host- and pathogen-related factors have been described in published cases. Diabetes mellitus has long been considered a predisposing factor for urinary tract infection, and multidrug-resistant epididymo-orchitis has been documented in diabetic patients, sometimes with devastating consequences [5]. Antimicrobial resistance among community uropathogens continues to rise globally, with first-line empirical oral therapy increasingly inadequate for complicated infections [2,5]. Recent genitourinary instrumentation, intravenous drug use and other risk factors that compromise host defenses have also been documented as antecedents in reported cases [5,7]. Delay between symptom onset and definitive imaging or surgery has been identified as a consistent contributor to testicular loss [4,6,7].

We describe a previously well diabetic 56-year-old man who progressed from clinical epididymo-orchitis to complete testicular infarction with tunica albuginea rupture despite outpatient antibiotic therapy, ultimately requiring orchiectomy. Microbiological culture confirmed a multidrug-resistant *Escherichia coli* sensitive only to carbapenems.

## Case presentation

**Figure 1:**
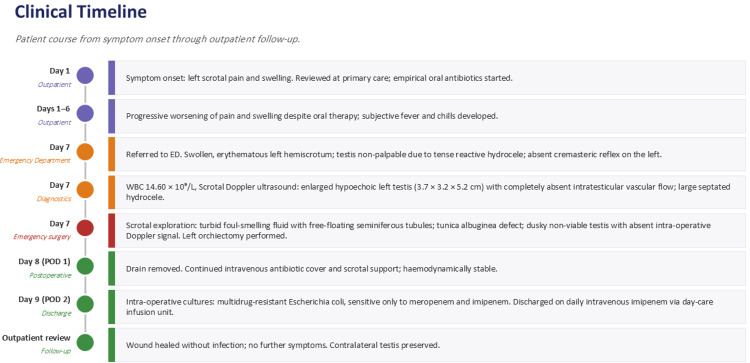
Clinical timeline summarizing the patient’s disease course from symptom onset through outpatient follow-up. The figure was generated using Microsoft PowerPoint.

A 56-year-old man with type 2 diabetes mellitus and no prior surgical history was referred from a primary care clinic in a remote village to our emergency department with a one-week history of left testicular pain and swelling. Symptoms had begun on the morning of presentation to his family physician, who started empirical oral antibiotic therapy on day 1. The exact outpatient antibiotic regimen was not available in the referral documentation and could not be reliably confirmed, as treatment had been initiated at an outside primary care facility. Pain and swelling progressed steadily over the following week and were accompanied by subjective fever and chills. He denied scrotal trauma, recent genitourinary instrumentation, urethral discharge, dysuria, ingestion of unpasteurized dairy, recent travel, or any prior similar episodes. He was sexually active with a single long-term partner. The full clinical course from symptom onset through outpatient follow-up is summarized in Figure 1.

**Figure 2:**
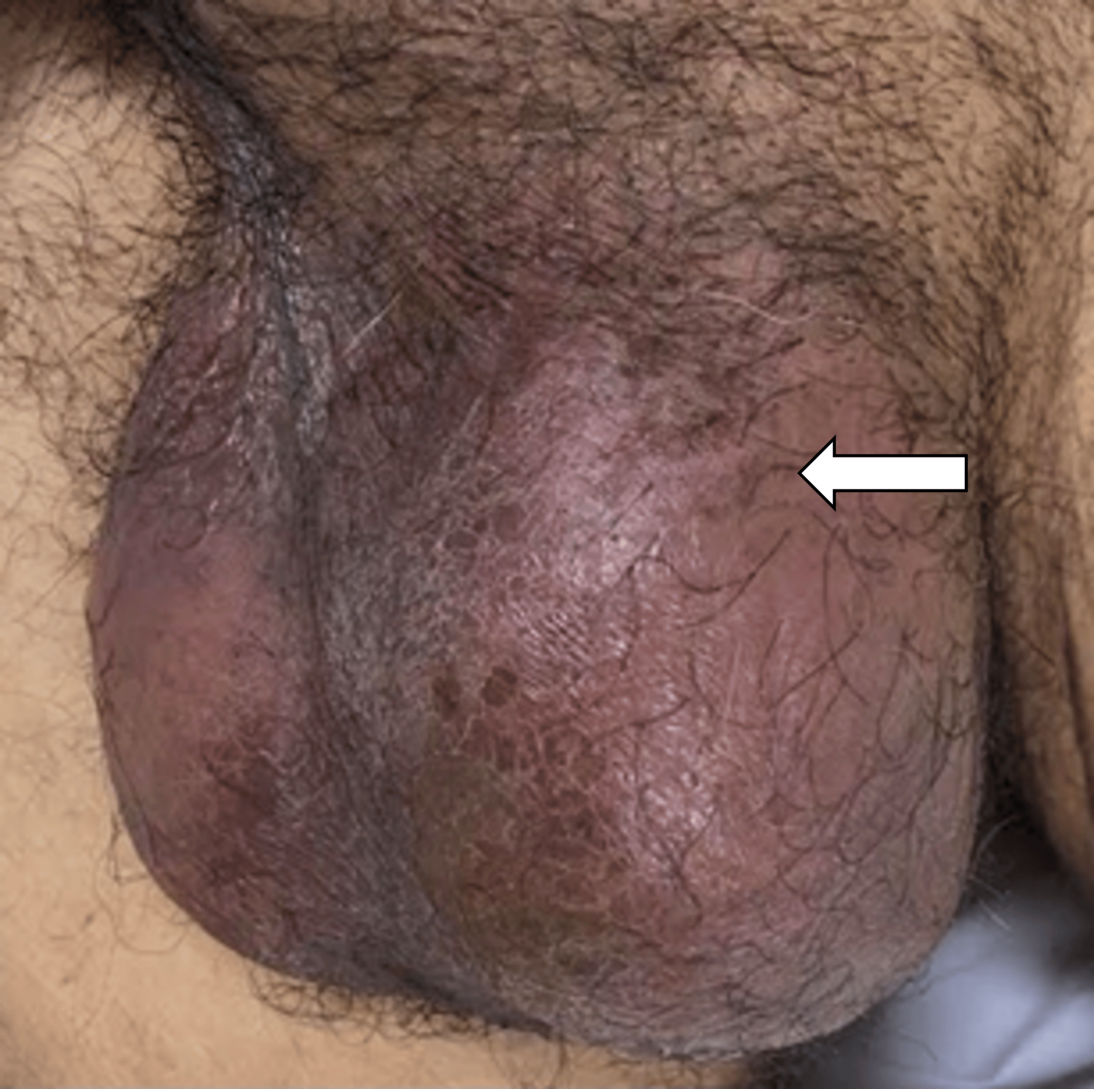
Clinical photograph at presentation showing a markedly swollen, erythematous left hemiscrotum (arrow). The right hemiscrotum is unremarkable.

On arrival, the patient was hemodynamically stable and afebrile. The left hemiscrotum was markedly swollen and erythematous with moderate tenderness; the testis itself could not be palpated due to a tense reactive hydrocele, although the spermatic cord was palpable. The cremasteric reflex was absent on the left side. The contralateral hemiscrotum was unremarkable, with a normally lying testis, no tenderness and an intact cremasteric reflex (Figure 2).

**Figure 3:**
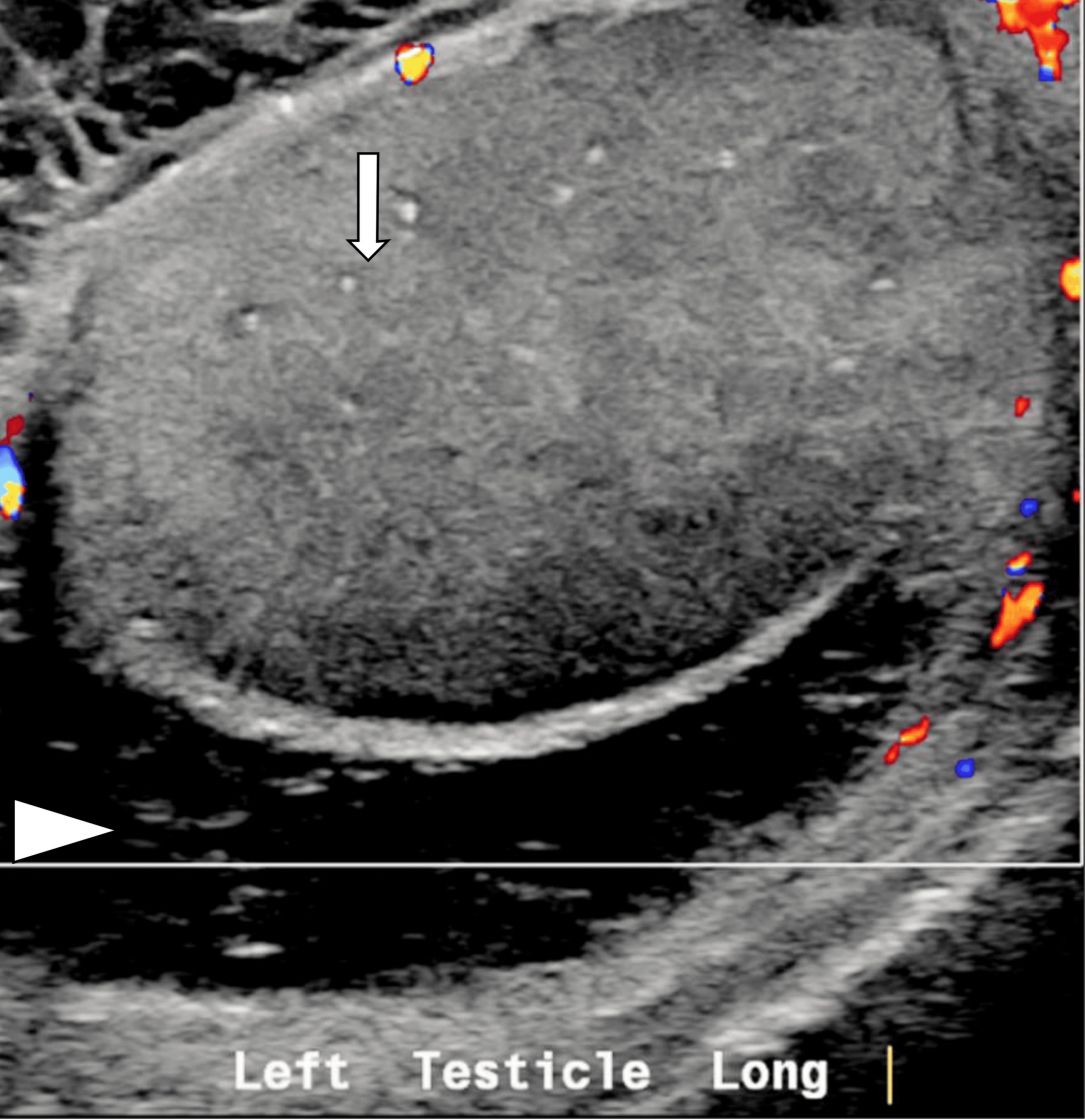
Scrotal ultrasound with color Doppler. The enlarged, diffusely hypoechoic left testis (arrow) measures 3.7 × 3.2 × 5.2 cm with completely absent intratesticular vascular flow with a large septated hydrocele (arrowhead).

Routine blood work and urinalysis were obtained on admission, with the principal results and reference ranges summarized in Table 1. The complete blood count demonstrated leukocytosis (14.60 × 10⁹/L) with neutrophilia (11.40 × 10⁹/L; 77.7%) consistent with active bacterial infection. The random plasma glucose was markedly elevated at 23.5 mmol/L, and a glycated hemoglobin (HbA1c) measured during the admission was 11.2%, indicating poorly controlled type 2 diabetes mellitus. Serum albumin was mildly low (33 g/L), consistent with an acute-phase response. Urinalysis demonstrated marked glucosuria and microscopic hematuria, but nitrites and leukocyte esterase were negative. Scrotal ultrasound with color Doppler demonstrated an enlarged, diffusely hypoechoic left testis measuring 3.7 × 3.2 × 5.2 cm with completely absent intratesticular vascular flow. There was a large septated left hydrocele and a congested spermatic cord. Findings were considered suggestive of testicular infarction, with the differential including a complication of an infectious process versus testicular torsion (Figure 3).

**Table 1:** Principal laboratory values at admission with reference ranges and units. H: above the reference range; L: below the reference range. †HbA1c was measured during hospitalization. Values are expressed in System International (SI) units; reference ranges are those of the reporting laboratory.

Parameter	Admission Value	Reference Range	Units
Hematology			
White blood cell count	14.60 (H)	4.0–11.0	× 10⁹/L
Neutrophils (absolute)	11.40 (H)	2.0–7.5	× 10⁹/L
Lymphocytes (absolute)	1.94	1.0–4.0	× 10⁹/L
Hemoglobin	139	130–180	g/L
Platelet count	303	150–400	× 10⁹/L
Renal profile			
Sodium	135	135–145	mmol/L
Potassium	3.5	3.5–5.0	mmol/L
Urea	4.1	2.5–7.5	mmol/L
Creatinine	63 (L)	64–110	µmol/L
Glycemic profile and serum protein			
Random plasma glucose	23.5 (H)	3.9–7.8	mmol/L
Glycated hemoglobin (HbA1c)†	11.2 (H)	4.0–6.0	%
Albumin	33 (L)	35–50	g/L
Urinalysis			
Glucose	≥1.000 (H)	Negative	g/L
Blood	0.03 (H)	Negative	–
Nitrites	Negative	Negative	–
Leukocyte esterase	Negative	Negative	–
White blood cells (urine)	1	0–5	/HPF
Red blood cells (urine)	4 (H)	0–2	/HPF

**Figure 4:**
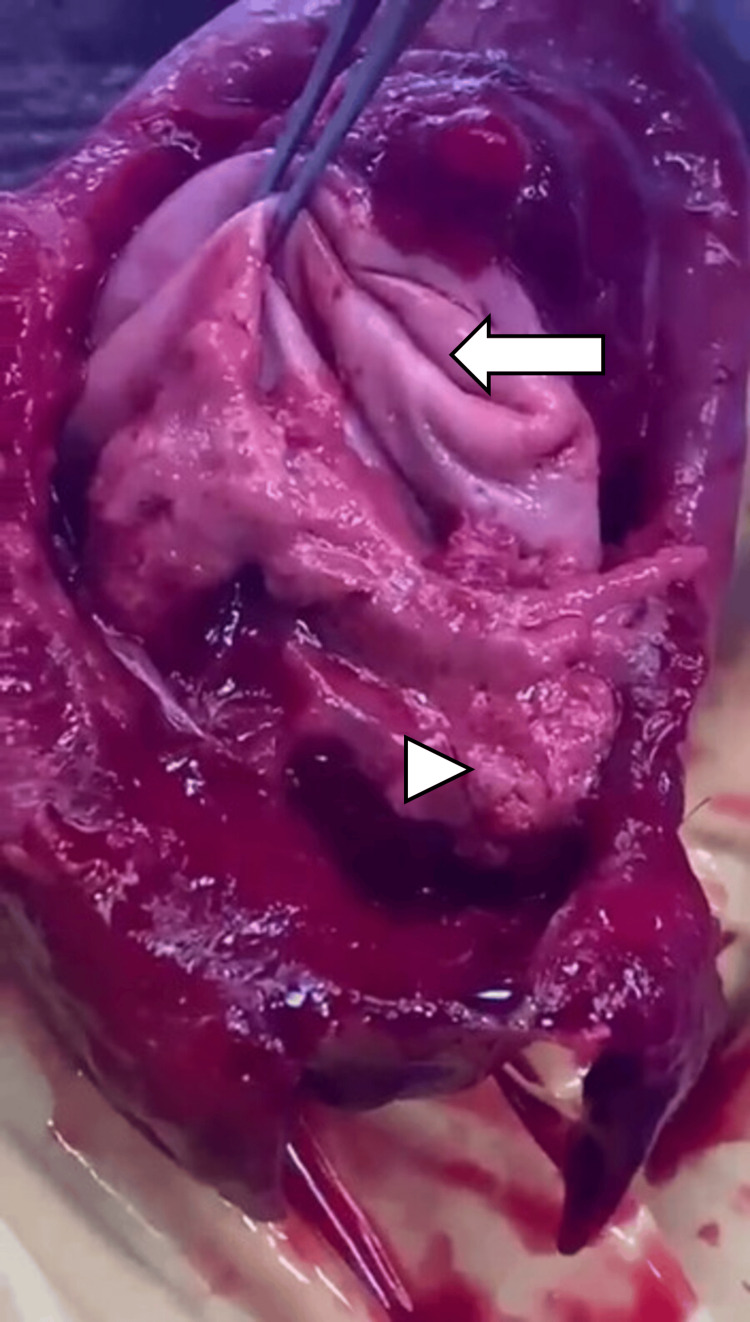
Intraoperative photograph showing the dusky, non-viable left testis (arrow) with a defect in the tunica albuginea and extruded seminiferous tubules (arrowhead), prior to orchiectomy.

The patient was taken urgently to the operating room. Under general anesthesia, after standard preparation and draping, a vertical left scrotal incision was made. Opening of the parietal tunica vaginalis released a gush of turbid, foul-smelling fluid containing free-floating seminiferous tubules; fluid was sent for microscopy, Gram stain and culture. The testis was delivered into the field and was found to have a defect in the tunica albuginea with extruded, dusky tubules. The spermatic cord was inspected intraoperatively, and no cord twist was identified. The operative findings were therefore more consistent with severe infective epididymo-orchitis complicated by global infarction and rupture rather than primary testicular torsion. Intraoperative Doppler ultrasound confirmed absent intratesticular flow. A left orchiectomy was therefore performed; the cord was secured with transfixation sutures of Vicryl 1-0 and PDS 1-0. Hemostasis was confirmed and a drain was left in situ. The dartos was closed with Vicryl 3-0 and the skin with Vicryl Rapide 3-0 (Figure 4).

The patient was transferred to the ward in stable condition with continued intravenous antibiotic cover and scrotal support. The drain was removed on postoperative day 1. Intraoperative cultures grew multidrug-resistant *Escherichia coli* sensitive only to meropenem and imipenem. Histopathology of the orchiectomy specimen demonstrated epididymo-orchitis with abscess formation, focal hemorrhage, edema, mixed inflammatory infiltrate, and extensive parenchymal necrosis. Unfortunately, pathology slides and gross pathology photographs were not available for inclusion. The patient was discharged on postoperative day 2 on daily intravenous imipenem to be administered in the day-care infusion unit. At outpatient review, the wound had healed without signs of infection, and he reported no further symptoms.

## Discussion

This case illustrates the worst-case trajectory of a common urological diagnosis: an apparently uncomplicated epididymo-orchitis ending in complete testicular infarction with tunica albuginea rupture in a middle-aged man with poorly controlled diabetes. Our findings, refractory progression despite outpatient empirical oral antibiotic therapy, an absent intratesticular Doppler signal on ultrasound, and a multidrug-resistant *Escherichia coli* on culture, mirror the small but growing case literature on this complication [4-9].

**Figure 5:**
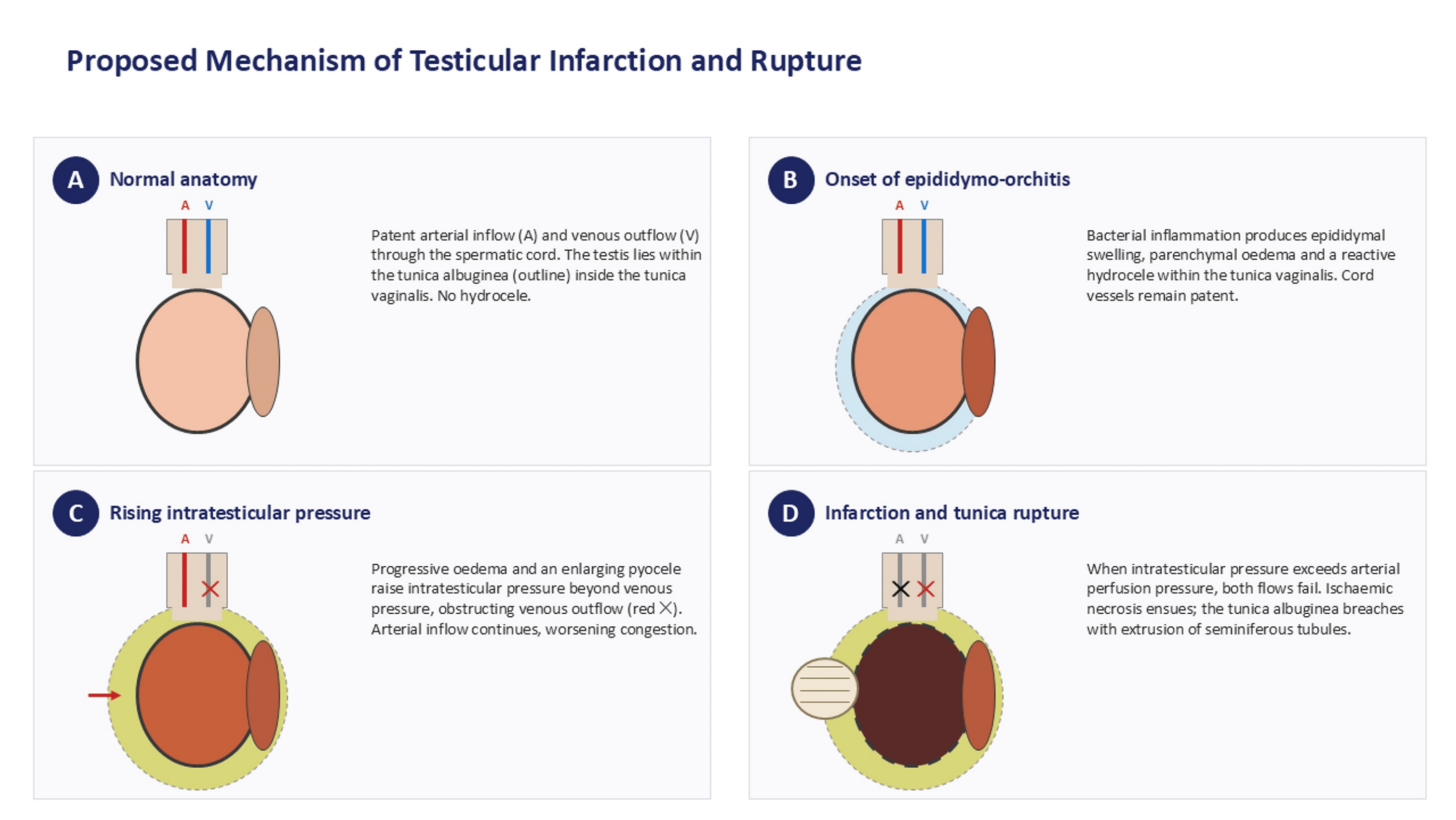
Schematic illustration of the proposed mechanism of testicular infarction and rupture as a complication of epididymo-orchitis. (A) Normal anatomy with patent arterial inflow (A) and venous outflow (V) through the spermatic cord. (B) Onset of epididymo-orchitis: bacterial inflammation produces epididymal swelling, parenchymal edema, and a reactive hydrocele within the tunica vaginalis. (C) Progressive edema and an enlarging pyocele raise intratesticular pressure beyond venous pressure, obstructing venous outflow (orange arrow). (D) When intratesticular pressure exceeds arterial perfusion pressure, ischemic necrosis ensues and the tunica albuginea breaches, with extrusion of seminiferous tubules. The figure was generated using Microsoft PowerPoint.

The pathophysiology proposed by Hourihane more than five decades ago and reinforced in subsequent series is one of inflammation-driven venous outflow obstruction: edema of the epididymis and spermatic cord, together with an enlarging pyocele, raises pressure within the closed tunica albuginea compartment beyond arterial perfusion pressure, producing ischemic infarction [9,10]. This sequence is depicted schematically in Figure 5.

This process may be conceptualized as a form of scrotal compartment syndrome. Similar to extremity compartment syndrome, progressive inflammatory edema and infected fluid accumulation within a relatively non-compliant compartment can first impair venous outflow and subsequently compromise arterial inflow.

Chia et al. described this pattern in a 50-year-old man whose initial Doppler study was reassuring but who subsequently developed both infarction and rupture, underscoring that a single reassuring early scan does not exclude the diagnosis [4]. Bilateral involvement, although exceptional, has also been reported and may result in functional castration [6]. Shen et al. additionally documented a case in which the tunica albuginea rupture led to spontaneous scrotal skin breach, emphasizing the destructive potential of unrecognized progression [7].

Differentiating impending infarction from uncomplicated epididymo-orchitis on clinical grounds alone is unreliable [4]. Scrotal Doppler ultrasound remains the diagnostic modality of choice and should be repeated whenever symptoms fail to improve on antibiotics or worsen during therapy [2]. The cardinal sonographic findings supporting infarction are heterogeneous testicular echotexture with absent or markedly reduced intratesticular vascular flow [5,6]. Reversal of diastolic flow in the testicular artery has been described as an early warning sign that may precede frank loss of perfusion and warrants close interval re-imaging [10]. Magnetic resonance imaging has occasionally been used in equivocal cases but are seldom required when Doppler is informative [6,11].

Several risk factors recur across reported cases. Diabetes mellitus, as in our patient, has long been considered a predisposing factor for genitourinary infection [12]. Our patient’s HbA1c of 11.2% indicates poorly controlled type 2 diabetes mellitus; while the relationship between glycemic control and uropathogen resistance has been variable across published series [12], multidrug-resistant epididymo-orchitis in diabetic patients has been documented [5]. Infection with resistant *Enterobacterales*, including extended-spectrum β-lactamase-producing or carbapenem-sensitive *Escherichia coli*, is increasingly encountered in clinical practice and may be underestimated when empirical therapy is initiated without culture data, particularly against a background of rising community antimicrobial resistance [2,5]. Delay between symptom onset and definitive imaging or surgery is a consistent contributor to testicular loss [4,6,7].

The relatively unremarkable admission urinalysis despite positive intraoperative culture is also noteworthy. This discrepancy may reflect prior outpatient antibiotic exposure partially sterilizing the urine while deep scrotal infection persisted, or localization of infection within a closed scrotal compartment with limited ongoing urinary shedding at the time of presentation.

Once Doppler demonstrates absent intratesticular flow, surgical exploration is needed both to exclude testicular torsion and to permit drainage, debridement, or orchiectomy depending on the operative findings [2,4,6]. Conservative management of segmental infarction has been reported in carefully selected patients with preserved adjacent perfusion [11], but global infarction with non-viable parenchyma and a ruptured tunica, as in this case, is not amenable to salvage. Postoperative antibiotic therapy must be tailored to culture and sensitivity results, with prolonged intravenous therapy frequently required when resistant organisms are identified [5].

The absent cremasteric reflex in this case represented an important diagnostic pitfall. Although loss of the cremasteric reflex is classically associated with testicular torsion, severe epididymo-orchitis with marked hemiscrotal edema, tense hydrocele or pyocele, and high local inflammatory pressure may clinically mimic torsion. Therefore, this sign should not be interpreted in isolation. In our patient, the absent reflex appropriately maintained torsion in the differential diagnosis and supported urgent exploration; however, the one-week infectious prodrome, turbid infected fluid, positive intraoperative culture, and absence of intraoperative evidence of cord torsion supported infection-related infarction rather than primary torsion.

Limitations of this report include the absence of pre-treatment microbiological data and the unavailable initial outpatient antibiotic regimen, as treatment was initiated at an outside facility before referral. Although histopathology confirmed epididymo-orchitis, pathology slides and gross pathology images were not available for inclusion. The short outpatient follow-up window also limits assessment of long-term endocrine, fertility, and quality-of-life outcomes. Finally, the single-case design limits generalizability.

## Conclusions

Testicular infarction with tunica albuginea rupture is a rare but devastating complication of epididymo-orchitis. In any patient, especially those with diabetes or other immunocompromising conditions, whose clinical course fails to improve on appropriate antibiotic therapy, urgent repeat scrotal Doppler ultrasound is warranted, and the absence of intratesticular flow demands immediate surgical exploration. Awareness of this trajectory, a low threshold for re-imaging, attention to local antimicrobial resistance patterns, and timely operative intervention together offer the only realistic prospects of preventing testicular loss and life-threatening infection.
